# Fission yeast Wee1 is required for stable kinetochore-microtubule attachment

**DOI:** 10.1098/rsob.230379

**Published:** 2024-01-03

**Authors:** Masahiro Takado, Takaharu G. Yamamoto, Yuji Chikashige, Tomohiro Matsumoto

**Affiliations:** ^1^ Radiation Biology Center, Graduate School of Biostudies, Kyoto University, Kyoto 606-8501, Japan; ^2^ Kobe Frontier Research Center, Advanced ICT Research Institute, National Institute of Information and Communications Technology, Kobe 651-2492, Japan

**Keywords:** Wee1, fission yeast, mitosis, spindle checkpoint, kinetochore

## Abstract

Wee1 is a cell cycle regulator that phosphorylates Cdk1/Cdc2 and inhibits G2/M transition. Loss of Wee1 in fission yeast results in an early onset of mitosis. Interestingly, we found that cells lacking Wee1 require the functional spindle checkpoint for their viability. Genetic analysis indicated that the requirement is not attributable to the early onset of mitosis. Live-cell imaging revealed that some kinetochores are not attached or bioriented in the *wee1* mutant. Furthermore, Mad2, a component of the spindle checkpoint known to recognize unattached kinetochores, accumulates in the vicinity of the spindle, representing activation of the spindle checkpoint in the mutant. It appears that the *wee1* mutant cannot maintain stable kinetochore-microtubule attachment, and relies on the delay imposed by the spindle checkpoint for establishing biorientation of kinetochores. This study revealed a role of Wee1 in ensuring accurate segregation of chromosomes during mitosis, and thus provided a basis for a new principle of cancer treatment with Wee1 inhibitors.

## Graphical abstract

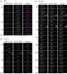

## 1. Introduction

Wee1 is a cell cycle regulator that coordinates the timing of the onset of mitosis with cell growth [1,2]. The term ‘wee’ was coined after fission yeast mutants with small cell size. They were initially identified through a genetic screen for *cdc* (cell division cycle) mutants [3], or for suppressors of the *cdc25-22* mutant that was arrested at the G2/M boundary at the restrictive temperature. The subsequent genetic analysis revealed that two genes, *wee1^+^* and *cdc2^+^*, are mutable to small-cell-sized mutants [4,5]. Extensive efforts have been made to elucidate the functional relationship among Wee1, Cdc2 and Cdc25 in fission yeast and other organisms, which have led to the conclusion as follows: Wee1 catalyses an inhibitory tyrosine phosphorylation of Cdc2/Cdk1, a cyclin-dependent kinase essential for G2/M transition. Cdc25 counteracts Wee1 by dephosphorylating Cdc2/Cdk1, driving the cell cycle into mitosis [6–8]. Although the fission yeast *wee* mutants allow for an earlier onset of mitosis and subsequent cell division, generating two smaller daughter cells, they are viable and no abnormal mitotic events have so far been reported.

Accurate chromosome segregation during mitosis is vital for stable transmission of genetic material. The spindle checkpoint is a surveillance mechanism that delays the onset of anaphase until all kinetochores are properly attached to the spindle, thus ensuring accurate chromosome segregation. The components of the spindle checkpoint accumulate at unattached kinetochores and form a complex, mitotic checkpoint complex (MCC) [9], transducing a signal to prevent activation of anaphase-promoting complex or cyclosome (APC/C), a specific E3 ubiquitin ligase [10,11]. Separase triggers sister chromatid separation by cleaving Scc1, a member of the cohesin complex. It is kept inactive during most of the cell cycle by binding securin, which is degraded following ubiquitination by APC/C [12]. The spindle checkpoint therefore prevents the onset of anaphase, the stage in which sister chromatids separate, by ultimately blocking separase until the last kinetochore is attached to the spindle.

In this report, we show that the *wee1* mutant requires the functional spindle checkpoint, whereas the *cdc2-1w* and *cdc2-3w* mutants, small-cell-sized mutants allelic to *cdc2^+^*, do not. Our analysis also shows that kinetochores are often unattached in the *wee1* mutant. Mad2, a component of MCC [9], accumulates in the vicinity of the spindle, presumably representing MCC assembled at an unattached kinetochore, and delays the onset of anaphase. We have thus concluded that the *wee1* mutant cannot maintain stable kinetochore-microtubule attachment, and relies on the delay imposed by the spindle checkpoint for establishing biorientation of kinetochores.

## 2. Results

### 2.1. The *wee1-50* mutant requires the functional spindle checkpoint

As shown in electronic supplementary material, figure S1A, we found that a *wee1-50 mad2Δ* (deletion for *mad2^+^*, a gene encoding a component of the spindle checkpoint) double mutant is lethal at 36°C. Likewise, introduction of deletion for other components of the checkpoint (Mad1 and Bub1) caused a temperature sensitivity of the growth in the *wee1-50* mutant (electronic supplementary material, figure S1A). Because the spindle checkpoint is a surveillance system that regulates the onset of anaphase [10,11], a stage well after the entry into mitosis, it was intriguing to pin down the underlying mechanism of the synergistic lethality between the loss of Wee1 and the spindle checkpoint.

Two alleles at the *cdc2^+^* locus, *cdc2-1w* and *cdc2-3w*, render Cdc2 insensitive to the negative control by Wee1 kinase. Keeping the Wee1 kinase functional, these alleles allow an earlier onset of mitosis [4,5]. As shown in electronic supplementary material, figure S1B, introduction of deletion for *mad2^+^* did not cause the lethality in the *cdc2-1w* and *cdc2-3w* mutants. The results along with other results from genetic studies (electronic supplementary material, figure S1C and table S2) suggested that the cause of the synergistic lethality is unrelated to the earlier onset of mitosis in the *wee1-50* mutant.

### 2.2. Kinetochore-microtubule attachment is not stable in the *wee1* mutants

Because the *wee1-50* mutant requires the functional spindle checkpoint for its viability, we attempted to identify an abnormal mitotic event in the mutant by time-lapse imaging of live cells. The spindle was fluorescently labelled by expressing α-tubulin tagged with mCherry and the kinetochores by Cnp1 (a fission yeast homologue of mammalian CENP-A) tagged with GFP.

**Figure 1.**
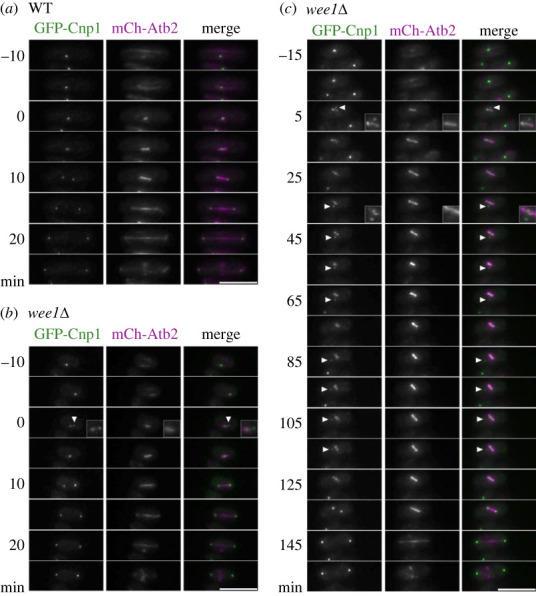
Kinetochore-microtubule attachment is not stable in the *wee1* mutants. Time-lapse images were taken every 5 min at 26°C. The Z-stack images were projected with a maximum intensity method. (*a*) An example of time-lapse images of the wild-type strain (WT) is shown. (*b*,*c*) Examples of the *wee1Δ* strain (*wee1Δ)*, in which the satellite kinetochore appeared once (*b*) and multiple times (*c*) during the observation, are shown. In the merged images, GFP-Cnp1 is shown in green, and mCh-Atb2 in magenta. The numbers on the left of the images indicate the time elapsed after the onset of mitosis. The arrowhead indicates the satellite kinetochore. The bar indicates 10 µm.

**Figure 2.**
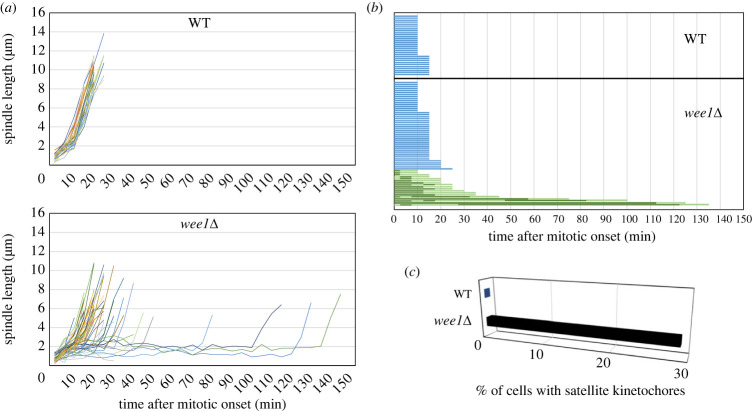
The onset of anaphase is delayed in the *wee1* mutants. The time-lapse images were analysed statistically for the wild-type strain (WT) (*n* = 30) and the *wee1Δ* strain (*n* = 62). (*a*) Kinetics of the spindle length in each of the WT and in the *wee1Δ* strain are shown graphically. (*b*) The mitotic duration (from the entry into mitosis to the onset of anaphase) for the WT and the *wee1Δ* strain (*wee1Δ*) is shown. The blue bars indicate cells with no satellite kinetochores. The light green bars indicate cells in which the satellite kinetochore appeared at least once, and the dark green bars the duration for which the satellite kinetochore was observed. (*c*) The percent of cells, in which satellite kinetochore appeared at least once, is shown.

Fission yeast centromeres/kinetochores are clustered and localized to the nuclear envelope near spindle pole body (SPB), a structure analogous to the centrosome. Upon the onset of mitosis, they are released from the nuclear envelope. Because all kinetochores are positioned close to SPB, they are quickly captured by microtubules and bioriented. Under a fluorescent microscope, they are therefore observed as a single speckle on the spindle [13,14]. Consistently, in each of the wild-type cells observed in this study, the single fluorescent speckle representing kinetochores was always found on the spindle from the onset of mitosis, which was defined by the disappearance of cytoskeletal microtubules and emergence of spindle microtubules, until the onset of anaphase (figure 1*a*). Mitosis progressed with no apparent delay and anaphase was initiated within 15 min from the entry into mitosis in the wild-type cells (figure 2*a*,*b*).

Time-lapse imaging analysis revealed abnormal positioning of kinetochores in the *wee1* (*wee1Δ*) mutant. The cluster of kinetochores frequently fell apart into a main cluster and a small ‘satellite kinetochore’ during mitosis (figure 1*b*). The satellite kinetochores eventually merged with the main cluster, but they occasionally reappeared (figure 1*c*). Because the kinetochores, which are captured and bioriented, are clustered and found on the spindle, the satellite kinetochores remained unattached, or detached from the spindle during the progression to anaphase in the *wee1* mutant (electronic supplementary material, figure S2). Importantly, the onset of anaphase was postponed until all the satellite kinetochores merged with the main cluster in the mutant (figure 2*a*,*b*). In approximately 29% (18 cells out of 62 cells observed) of the *wee1* mutants, the satellite kinetochore appeared at least once during mitosis (figure 2*c*).

It has been reported that dikaryotic cells spontaneously emerge in the *wee1* mutant [4,15], though the precise mechanism remains unknown. Consistently, we observed 76 *wee1Δ* cells, of which 14 cells were dikaryotic. We excluded these cells from the statistical analysis because we were unable to accurately identify satellite kinetochores (electronic supplementary material, figure S3).

### 2.3. The spindle checkpoint is activated in the *wee1* mutants

We speculated that the delay in the onset of anaphase observed in the *wee1* mutant was imposed by the spindle checkpoint. In order to confirm the activation of the checkpoint, we monitored Mad2, a component of the checkpoint shown to accumulate on unattached kinetochores [16–18]. The spindle was fluorescently labelled by expressing α-tubulin tagged with mCherry and Mad2 tagged with GFP.

**Figure 3.**
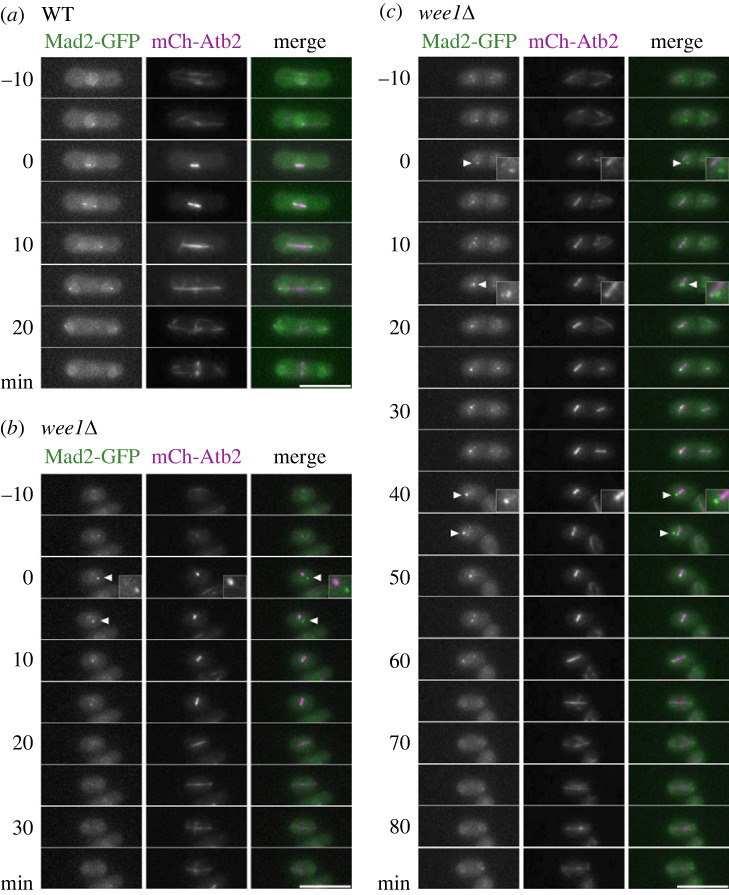
The spindle checkpoint is activated in the *wee1* mutants. Time-lapse images were taken every 5 min at 26°C. The Z-stack images were projected with a maximum intensity method. Examples of time-lapse images of the wild-type strain (WT) (*a*) and the *wee1Δ* strain (*wee1Δ)* (*b*,*c*) are shown. Bright Mad2-GFP speckle appeared once (*b*) or multiple times (*c*) in the *wee1Δ* strain. In the merged images, Mad2-GFP is shown in green and mCh-Atb2 in magenta. The numbers on the left of the images indicate the time elapsed after the onset of mitosis. The arrowhead indicates the Mad2 speckle. The bar indicates 10 µm.

**Figure 4.**
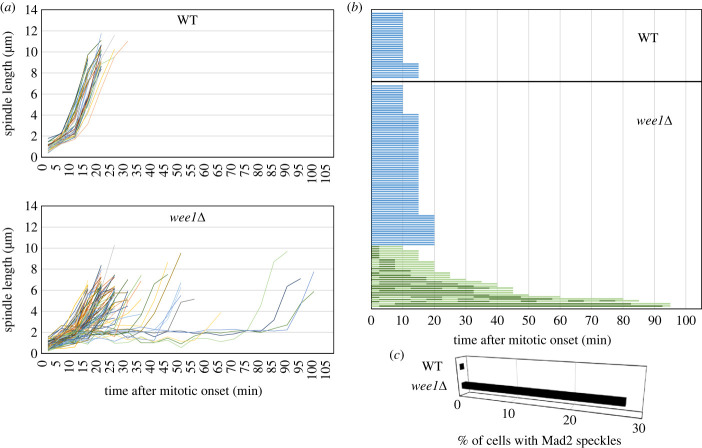
The activation of spindle checkpoint delays anaphase onset in the *wee1* mutants. (*a*) The time-lapse images were analysed statistically for the wild-type strain (WT) (*n* = 30) and the *wee1Δ* strain (*n* = 101). Kinetics of the spindle length in each of the WT and in the *wee1Δ* strain are shown graphically. (*b*) The mitotic duration (from the entry into mitosis to the onset of anaphase) for the WT and the *wee1Δ* strain (*wee1Δ*) is shown. The blue bars indicate cells with no Mad2 speckles. The light green bars indicate cells in which Mad2 speckle appeared at least once, and the dark green bars the duration for which Mad2 speckle was observed. (*c*) The percent of cells, in which Mad2 speckle appeared at least once, is shown.

Consistently with previous study [19,20], Mad2 was observed as a single speckle, likely at SPB or the cluster of kinetochores around the onset of mitosis, suggesting that SPB may serve as a reservoir of Mad2, or kinetochores clustering near SPB are not attached to the spindle. As mitosis progressed, it was faintly found on the entire length of the spindle and SPB in the wild-type cells (figure 3*a*). By contrast, Mad2 was found as one or two bright speckles in the vicinity of the spindle during mitosis in the *wee1* mutant (figure 3*b*,*c*). Judged by the length of the spindle, anaphase was initiated soon after Mad2 speckles disappeared (figure 4*a*,*b*). In approximately 28% (28 cells out of 101 cells observed) of the *wee1* mutants, the Mad2 speckle appeared at least once (figure 4*c*). The observation thus suggested that the spindle checkpoint was activated in the *wee1* mutants.

**Figure 5.**
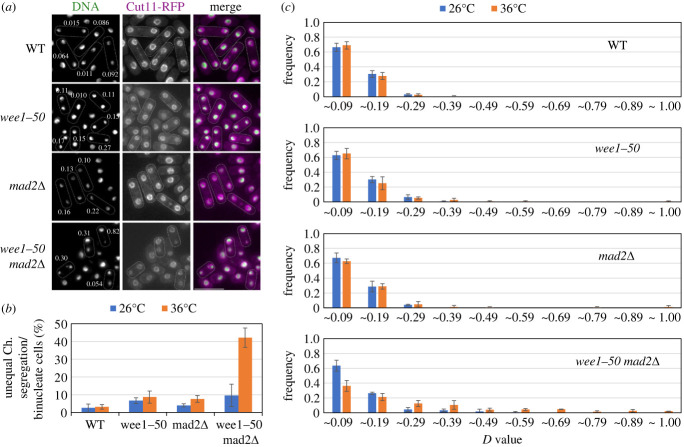
Unequal chromosome segregation in the *wee1-50* mutant lacking Mad2. (*a*) The wild-type strain (WT) and three mutants, *wee1-50*, *mad2Δ* and *wee1-50 mad2Δ* expressing Cut11 tagged with RFP (a marker for the nuclear membrane, used to identify binucleated cells in which the two nuclei were on the same focal plane), were grown at 26°C and then shifted to 36°C for 1 h. Difference in the nuclear size between the two nuclei in a binucleated cell (D value) was determined as described in Materials and Methods. Binucleated cells, surrounded by the dotted line, are shown with the D value. The bar indicates 10 µm. (*b*) Assuming that the size of a nucleus is roughly proportional to its DNA content, we used the D value to identify cells which had undergone unequal chromosome segregation. The percentage of binucleated cells with D value greater than 0.2 is shown. (*c*) The distribution of the D value is shown.

The results described above indicated that the *wee1* mutant cannot maintain stable kinetochore-microtubule attachment, and heavily relies on the delay imposed by spindle checkpoint for reestablishing biorientation of kinetochores. As shown in figure 5, the *wee1* mutants with no functional spindle checkpoint indeed failed in accurate chromosome segregation and generated two sister cells with unequal nuclear size.

## 3. Discussion

In this study, by live cell imaging analysis, we showed that kinetochores are frequently unattached and the onset of anaphase is delayed in the *wee1* mutant. We confirmed that the delay in the onset of anaphase in the *wee1* mutant is imposed by the spindle checkpoint that is activated by the accumulation of Mad2, representing formation of MCC for signalling of the checkpoint. Although not directly demonstrated in this study, we speculate that Mad2 accumulates as the speckle(s) at unattached kinetochores in the vicinity of the spindle in the *wee1* mutant. Based on these results, we propose that the *wee1* mutant is unable to maintain stable kinetochore-microtubule attachment. It is likely that the activity of Wee1 kinase is necessary for stabilizing this attachment.

In addition to Cdks, at least two other proteins have been characterized, to our knowledge, as substrates of Wee1 kinase. KLP61F, a kinesin-5 in *Drosophila*, is phosphorylated by Wee1 at three tyrosine residues (Y23, Y152 and Y207) within the head domain. The expression of KLP61F^3YF^, a mutant carrying replacement of the three tyrosine residues with phenylalanines, causes abnormal morphology of the spindle. The phenotypes have been observed in embryos lacking Wee1, and shown not to be due to premature entry into mitosis [21,22]. Among the three tyrosine residues of KLP61F, Y207 is conserved in the human kinesin-5 (Eg5) at Y211, which is phosphorylated by Src kinase, not Wee1. Replacement of Y211 with a phosphomimetic amino acid (glutamic acid) leads to the formation of monopolar spindles *in vivo*, a phenotype observed in cells lacking endogenous Eg5. Replacement of Y211 with phenylalanine causes disorganized spindles, which mimics the effect of the Src kinase inhibitor, SU6566 [23]. This suggests that Y211 in Eg5 and likely Y207 in KLP61F are major tyrosine residues that regulate kinesin-5 through phosphorylation. Cut7 is the only known kinesin-5 in fission yeast. The function of Wee1 in controlling Cut7 is not clear because Cut7 lacks a tyrosine residue at the position corresponding to Y207 in *Drosophila* KLP61F. Wee1 also phosphorylates histone H2B at Tyr37 in mammalian cells and at Tyr40 in budding yeast. The phosphorylation of Tyr37 occurs upstream of histone cluster, Hist1, and inhibits the transcription of multiple histone genes [24]. The Tyr37 in histone H2B is conserved in fission yeast, but it has not been thoroughly studied. Currently, we do not have strong evidence to suggest that the two proteins, Cut7 and histone H2B, are not regulated by Wee1 kinase in order to maintain stable kinetochore-microtubule attachment. It is, however, plausible to speculate that Wee1 phosphorylates and regulates a protein more directly involved in kinetochore assembly, capturing kinetochores or a relating process.

Wee1 has been extensively studied as a therapeutic target for cancer treatment. Inhibition of Wee1 is expected to force cancer cells undergoing DNA replication or repair to enter mitosis, resulting in cell death through mitotic catastrophe [25–27]. Interestingly, two studies have reported that a Wee1 inhibitor, MK-1775, causes a delay in mitotic progression. In HeLa cells treated with the inhibitor at prometaphase, the onset of anaphase is significantly delayed [28]. Another study has shown that human tongue squamous carcinoma cells (SAS) treated with MK-1775 are arrested at metaphase. The arrest is dependent on the spindle checkpoint because it is abrogated by an inhibitor of Mps1, an essential kinase of the checkpoint signalling [29]. Our study presented here suggests that the Wee1 inhibitor MK-1775 may act by destabilizing the attachment between kinetochores and microtubules, and could serve as a basis for improving and developing Wee1-targeted cancer chemotherapy.

## Data Availability

Supplementary material is available online [30].

## References

[1] Kellogg DR. 2003 Wee1-dependent mechanisms required for coordination of cell growth and cell division. J. Cell Sci. **116**, 4883-4890. (10.1242/jcs.00908) 14625382

[2] Wood E, Nurse P. 2015 Sizing up to divide: mitotic cell-size control in fission yeast. Annu. Rev. Cell Dev. Biol. **31**, 11-29. (10.1146/annurev-cellbio-100814-125601) 26566110

[3] Nurse P. 1975 Genetic control of cell size at cell division in yeast. Nature **256**, 547-551. (10.1038/256547a0) 1165770

[4] Thuriaux P, Nurse P, Carter B. 1978 Mutants altered in the control co-ordinating cell division with cell growth in the fission yeast Schizosaccharomyces pombe. Mol. Gen. Genet. **161**, 215-220. (10.1007/BF00274190) 672898

[5] Fantes PA. 1981 Isolation of cell size mutants of a fission yeast by a new selective method: characterization of mutants and implications for division control mechanisms. J. Bacteriol. **146**, 746-754. (10.1128/jb.146.2.746-754.1981) 7217015 PMC217021

[6] Russell P, Nurse P. 1987 Negative regulation of mitosis by wee1+, a gene encoding a protein kinase homolog. Cell **49**, 559-567. (10.1016/0092-8674(87)90458-2) 3032459

[7] Nurse P. 1990 Universal control mechanism regulating onset of M-phase. Nature **344**, 503-508. (10.1038/344503a0) 2138713

[8] Coleman TR, Dunphy WG. 1994 Cdc2 regulatory factors. Curr. Opin. Cell Biol. **6**, 877-882. (10.1016/0955-0674(94)90060-4) 7880537

[9] Sudakin V, Chan GK, Yen TJ. 2001 Checkpoint inhibition of the APC/C in HeLa cells is mediated by a complex of BUBR1, BUB3, CDC20, and MAD2. J. Cell Biol. **154**, 925-936. (10.1083/jcb.200102093) 11535616 PMC2196190

[10] Zich J, Hardwick KG. 2010 Getting down to the phosphorylated 'nuts and bolts' of spindle checkpoint signalling. Trends Biochem. Sci. **35**, 18-27. (10.1016/j.tibs.2009.09.002) 19836959

[11] Musacchio A. 2015 The molecular biology of spindle assembly checkpoint signaling dynamics. Curr. Biol. **25**, R1002-R1018. (10.1016/j.cub.2015.08.051) 26485365

[12] Peters JM. 2002 The anaphase-promoting complex: proteolysis in mitosis and beyond. Mol. Cell **9**, 931-943. (10.1016/s1097-2765(02)00540-3) 12049731

[13] Nabeshima K, Nakagawa T, Straight AF, Murray A, Chikashige Y, Yamashita YM, Hiraoka Y, Yanagida M. 1998 Dynamics of centromeres during metaphase-anaphase transition in fission yeast: Dis1 is implicated in force balance in metaphase bipolar spindle. Mol. Biol. Cell **9**, 3211-3225. (10.1091/mbc.9.11.3211) 9802907 PMC25611

[14] Hirai H, Arai K, Kariyazono R, Yamamoto M, Sato M. 2014 The kinetochore protein Kis1/Eic1/Mis19 ensures the integrity of mitotic spindles through maintenance of kinetochore factors Mis6/CENP-I and CENP-A. PLoS ONE **9**, e111905. (10.1371/journal.pone.0111905) 25375240 PMC4222959

[15] Grallert A, Grallert B, Ribar B, Sipiczki M. 1998 Coordination of initiation of nuclear division and initiation of cell division in Schizosaccharomyces pombe: genetic interactions of mutations. J. Bacteriol. **180**, 892-900. (10.1128/JB.180.4.892-900.1998) 9473044 PMC106969

[16] Chen RH, Waters JC, Salmon ED, Murray AW. 1996 Association of spindle assembly checkpoint component XMAD2 with unattached kinetochores. Science **274**, 242-246. (10.1126/science.274.5285.242) 8824188

[17] Li Y, Benezra R. 1996 Identification of a human mitotic checkpoint gene: hsMAD2. Science **274**, 246-248. (10.1126/science.274.5285.246) 8824189

[18] Waters JC, Chen RH, Murray AW, Salmon ED. 1998 Localization of Mad2 to kinetochores depends on microtubule attachment, not tension. J. Cell Biol. **141**, 1181-1191. (10.1083/jcb.141.5.1181) 9606210 PMC2137189

[19] Ikui AE, Furuya K, Yanagida M, Matsumoto T. 2002 Control of localization of a spindle checkpoint protein, Mad2, in fission yeast. J. Cell Sci. **115**, 1603-1610. (10.1242/jcs.115.8.1603) 11950879

[20] Saitoh S, Ishii K, Kobayashi Y, Takahashi K. 2005 Spindle checkpoint signaling requires the mis6 kinetochore subcomplex, which interacts with mad2 and mitotic spindles. Mol. Biol. Cell **16**, 3666-3677. (10.1091/mbc.e05-01-0014) 15930132 PMC1182306

[21] Stumpff J, Kellogg DR, Krohne KA, Su TT. 2005 Drosophila Wee1 interacts with members of the gammaTURC and is required for proper mitotic-spindle morphogenesis and positioning. Curr. Biol. **15**, 1525-1534. (10.1016/j.cub.2005.07.031) 16139207 PMC3242738

[22] Garcia K, Stumpff J, Duncan T, Su TT. 2009 Tyrosines in the kinesin-5 head domain are necessary for phosphorylation by Wee1 and for mitotic spindle integrity. Curr. Biol. **19**, 1670-1676. (10.1016/j.cub.2009.08.013) 19800237 PMC2762001

[23] Bickel KG, Mann BJ, Waitzman JS, Poor TA, Rice SE, Wadsworth P. 2017 Src family kinase phosphorylation of the motor domain of the human kinesin-5, Eg5. Cytoskeleton (Hoboken) **74**, 317-330. (10.1002/cm.21380) 28646493 PMC5735839

[24] Mahajan K, Fang B, Koomen JM, Mahajan NP. 2012 H2B Tyr37 phosphorylation suppresses expression of replication-dependent core histone genes. Nat. Struct. Mol. Biol. **19**, 930-937. (10.1038/nsmb.2356) 22885324 PMC4533924

[25] Do K, Doroshow JH, Kummar S. 2013 Wee1 kinase as a target for cancer therapy. Cell Cycle **12**, 3159-3164. (10.4161/cc.26062) 24013427 PMC3865011

[26] Matheson CJ, Backos DS, Reigan P. 2016 Targeting WEE1 kinase in cancer. Trends Pharmacol. Sci. **37**, 872-881. (10.1016/j.tips.2016.06.006) 27427153

[27] da Costa AABA, Chowdhury D, Shapiro GI, D'Andrea AD, Konstantinopoulos PA. 2023 Targeting replication stress in cancer therapy. Nat. Rev. Drug Discov. **22**, 38-58. (10.1038/s41573-022-00558-5) 36202931 PMC11132912

[28] Lewis CW, Jin Z, Macdonald D, Wei W, Qian XJ, Choi WS, He R, Sun X, Chan G. 2017 Prolonged mitotic arrest induced by Wee1 inhibition sensitizes breast cancer cells to paclitaxel. Oncotarget **8**, 73 705-73 722. (10.18632/oncotarget.17848) PMC5650293 29088738

[29] Nojima H, Homma H, Onozato Y, Kaida A, Harada H, Miura M. 2020 Differential properties of mitosis-associated events following CHK1 and WEE1 inhibitor treatments in human tongue carcinoma cells. Exp. Cell Res. **386**, 111720. (10.1016/j.yexcr.2019.111720) 31738907

[30] Takado M, Yamamoto TG, Chikashige Y, Matsumoto T. 2023 Fission yeast Wee1 is required for stable kinetochore-microtubule attachment. Figshare. (10.6084/m9.figshare.c.6960424)38166399

